# Proprotein convertase subtilisin/kexin type 9 (PCSK9) in Alzheimer’s disease: A genetic and proteomic multi-cohort study

**DOI:** 10.1371/journal.pone.0220254

**Published:** 2019-08-22

**Authors:** Cynthia Picard, Alexandre Poirier, Stéphanie Bélanger, Anne Labonté, Daniel Auld, Judes Poirier, on behalf of the PREVENT-AD Research Group

**Affiliations:** 1 Douglas Mental Health University Institute, Montréal, Québec, Canada; 2 Centre for the Studies in the Prevention of Alzheimer’s Disease, Montréal, Québec, Canada; 3 Department of Psychiatry, McGill University, Montréal, Québec, Canada; 4 Génome Québec Innovation Centre, Montréal, Québec, Canada; Torrey Pines Institute for Molecular Studies, UNITED STATES

## Abstract

**Background:**

Proprotein convertase subtilisin/kexin type 9 (PCSK9) is a hepatic enzyme that regulates circulating low-density lipoprotein (LDL) cholesterol levels by binding to LDL receptors (LDLR) and promoting their degradation. Although PCSK9 inhibitors were shown to reduce the risk of cardiovascular disease, a warning was issued concerning their possible impact on cognitive functions. In Alzheimer’s disease (AD), it is believed that cognitive impairment is associated with cholesterol metabolism alterations, which could involve PCSK9. The main objective of this study is to determine if PCSK9 plays a significant role in the pre-symptomatic phase of the disease when the pathophysiological markers of AD unfolds and, later, when cognitive symptoms emerge.

**Methods and findings:**

To test if *PCSK9* is associated with AD pathology, we measured its expression levels in 65 autopsy confirmed AD brains and 45 age and gender matched controls. Messenger ribonucleic acid (mRNA) were quantified using real-time polymerase chain reaction (RT-PCR) and protein levels were quantified using enzyme-linked immunosorbent assay (ELISA). *PCSK9* was elevated in frontal cortices of AD subjects compared to controls, both at the mRNA and protein levels. LDLR protein levels were unchanged in AD frontal cortices, despite and upregulation at the mRNA level. To verify if PCSK9 dysregulation was observable before the onset of AD, we measured its expression in the cerebrospinal fluid (CSF) of 104 “at-risk” subjects and contrasted it with known apolipoproteins levels and specific AD biomarkers using ELISAs. Positive correlations were found between CSF PCSK9 and apolipoprotein E (APOE), apolipoprotein J (APOJ or CLU), apolipoprotein B (APOB), phospho Tau (pTau) and total Tau. To investigate if *PCSK9* levels were driven by genetic variants, we conducted an expression quantitative trait loci (eQTL) study using bioinformatic tools and found two polymorphisms in strong association. Further investigation of these variants in two independent cohorts showed a female specific association with AD risk and with CSF Tau levels in cognitively impaired individuals.

**Conclusions:**

*PCSK9* levels differ between control and AD brains and its protein levels correlate with those of other lipoproteins and AD biomarkers even before the onset of the disease. PCSK9 regulation seems to be under tight genetic control in females only, with specific variants that could predispose to increased AD risk.

## Introduction

Statin therapy [1] and the PCSK9 inhibitor evolocumab [2] have been shown to lower LDL cholesterol levels and to reduce the rate of cardiovascular events among patients with established cardiovascular disease. Post marketing surveillance reports and some randomized trials of statin therapy [3, 4] have suggested that statins, or the associated low levels of LDL cholesterol, may be associated with impaired cognitive function, which led the Food and Drug Administration to issue a warning in 2012. More recently, two clinical trials of PCSK9 inhibitors and a meta-analysis showed a possible association between these drugs and cognitive adverse advents [5, 6]. In contrast, a large randomized trial involving patients who received either PCSK9-evolocumab or placebo in addition to statin therapy revealed no significant between-group difference in cognitive function over the course of 18 months [7]. The interplay between lipid homeostasis and cognition has been the focus of attention of many fields of research, particularly in dementing illnesses.

PCSK9 is a critical regulator of LDL-cholesterol metabolism which acts as an endogenous inhibitor of the LDL receptor (LDLR) pathway. Briefly, after intracellular autocleavage, PCSK9 is secreted and binds to the extracellular domain of the LDLR family members at the cell surface of neurons and glial cells and targets the receptors for lysosomal degradation [8]. PCSK9 was shown to downregulate LDLR levels during brain development in mice whereas *Pcsk9* knockout mice did not show signs of altered central nervous system (CNS) development [9, 10]. A more controversial issue is the role of PCSK9 on amyloid deposition and AD. PCSK9 has been shown to modulate the degradation of BACE1 (beta-site amyloid precursor protein cleaving enzyme 1), the enzyme involved in the generation of the amyloid beta-peptides 1–40 and 1–42 [11] with increased levels of BACE1 and total amyloid beta in the brain of *Pcsk9* knockout mice: results that were, however, not confirmed in a subsequent study [12].

Altered lipid metabolism has been extensively implicated in late onset Alzheimer’s disease (LOAD) pathogenesis but the molecular basis of this relationship is not well understood. Yet, the association between lipoprotein metabolism and LOAD is best exemplified by the candidate risk/protective genes identified to date in genome wide association studies. The “top 15” genetic risk factors include *APOE* and *APOJ* (or *CLU*) involved in extracellular cholesterol transport; *BIN1*, *SORL1* and *PICALM*, regulating cholesterol-rich lipoprotein internalization; and *ABCG1* and *ABCA7* actively involved in intracellular cholesterol transport [13, 14]. Recently, we reported a significant risk reduction for LOAD mediated by the rs3846662 “AA” single nucleotide polymorphism (SNP) in the *HMGCR* gene coding for the 3-hydroxy-3-methylglutaryl-CoA reductase, the rate limiting step for cholesterol synthesis in the central nervous system (CNS) and the pharmacological target of statin therapies [15, 16]. We also showed neuroprotection for LOAD associated with the T allele of rs2269657 in *SREBF2* gene [17], coding for a transcription factor involved in the regulation of *HMGCR* [18], *LDLR* [19] and *PCSK9* [20].

At the molecular level, pharmacological inhibition of cholesterol synthesis in the brain by statins markedly reduced the production of amyloid beta (Aβ) in wild type mouse and guinea pigs whereas, inhibitors of cholesterol esterification (storage), markedly increase Aβ synthesis [21–23]. More interestingly, chronic administration of cholesterol synthesis inhibitors (statins) in transgenic mice over expressing the amyloid beta precursor protein (APP) significantly reduces Aβ production and the accumulation of amyloid plaques in the brain during aging [24].

Epidemiological studies have shown clear associations between high cholesterol levels in midlife and LOAD risk [25–27] whereas cholesterol lowering drugs have been shown to have a protective effect against the development of dementia in retrospective [28–30] and prospective studies [31, 32]. However, randomized control trials in symptomatic LOAD subjects found no cognitive benefit of statin treatments [33, 34]. The most recent prospective epidemiological studies in which statins were stratified as water or lipid soluble report significant protection in users of the latter category [31, 32]; consistent with improved brain barrier penetration.

These observations prompted us to examine more closely the neurobiology of PCSK9 in autopsied normal and diseased LOAD brains as well as, in the cerebrospinal fluid (CSF) of aged cognitively normal but “at-risk” subjects with a parental history of LOAD. The main objective of this study was to determine if PCSK9, a very potent modulator of cholesterol metabolism seldom associated with cognitive dysfunction, plays a significant role in the pre-symptomatic phase of the disease when the pathophysiological markers of LOAD unfolds and, later, when cognitive symptoms emerge.

## Material and methods

### Participant samples

Participants from three different cohorts with available samples are described in Table 1.

**Table 1:** Description of the three cohorts.

	PREVENT-AD	QFP				ADNI			
Samples	DNA + CSF	DNA (n = 1952)		Brains (n = 110)		DNA (n = 535)		CSF (n = 384)	
Groups	CTL (n = 104)	CTL (n = 986)	AD (n = 966)	CTL (n = 45)	AD (n = 65)	CTL (n = 195)	AD (n = 340)	CTL (n = 109)	MCI/AD (n = 275)
APOE4+	38%	18%	50%	33%	65%	26%	67%	24%	59%
Females	70%	73%	65%	42%	51%	47%	43%	48%	37%
Age ±SD	63 ±6 y.o.	≥65 y.o.	≥65 y.o.	70 ±12 y.o.	78 ±9 y.o.	76 ±5 y.o.	75 ±7 y.o.	75 ±5 y.o.	75 ±8 y.o.
BMI ±SD	27 ±5	N/A	N/A	N/A	N/A	26 ±4	26 ±4	27 ±5	26 ±4
Chol ±SD	207 ±33*	N/A	N/A	N/A	N/A	194 ±41	198 ±40	192 ±38	198 ±43
TG ±SD	170 ±101*	N/A	N/A	N/A	N/A	141 ±81	154 ±89	142 ±92	163 ±158
MOCA ±SD	28 ±2	N/A	N/A	N/A	N/A	25 ±3**	14 ±6**	25 ±3**	18 ±6**

AD, Alzheimer’s disease; ADNI, Alzheimer’s disease Neuroimaging Initiative; APOE4, apolipoprotein E (ε4 allele); BMI, body mass index; Chol, cholesterol; CSF, cerebrospinal fluid; CTL, controls; MCI, mild cognitive impairment; MOCA, Montreal Cognitive Assessment; PREVENT-AD, pre-symptomatic evaluation of experimental or novel treatments for Alzheimer’s disease; QFP, Quebec founder population; SD, standard deviation; TG, triglycerides.

*non-fasting

**not performed at baseline

### Brain tissues

Frozen human frontal cortices and cerebella specimens from 65 LOAD cases and 45 controls were used for mRNA and protein expression studies. All subjects originated from the same eastern Canadian population and donated their brains to the Douglas—Bell Canada Brain Bank in Montreal. Neuropathological analyses were consistent with the criteria used in the classification of Khachaturian [35] and were defined as sporadic LOAD because family history did not reveal any first-degree relative with LOAD. Age-matched autopsy confirmed control cases were free of brain neuropathological lesions (neurofribillary tangles and senile plaques < 20/mm^3^ and < 10/mm^3^, respectively). This study is conformed to The Code of Ethics of the World medical Association and was approved by the Ethics Board of the Douglas Hospital Research Center. All participants signed an informed consent.

### Real-time polymerase chain reaction (RT-PCR)

Total ribonucleic acid (RNA) was isolated from frozen brain tissues using the Maxwell 16 Tissue LEV total RNA Purification kit and Maxwell 16 instrument (Promega, Madison, WI). Purity and quality of each RNA extract was analysed by spectrophotometric A260nm/A280nm ratio and by Bioanalyser (Agilent Technologies, Palo Alto, CA) according to the manufacturer’s guidelines. The reverse transcription (RT) reaction was performed with the high capacity cDNA RT kit (Applied Biosystems, Foster City, CA) on a Multigene thermal cycler (Labnet International Inc., Woodbridge, NJ). Total RNA (500 ng) was used for each 50 μl RT_PCR reaction. RT-PCR was performed with a 7500 Fast Real-Time PCR System (Applied Biosystems) with assay-on-demand gene expression product for *PCSK9* (Hs00545399_m1) and low-density lipoprotein receptor (*LDLR*) (Hs01092524_m1) as target genes and hypoxanthine phosphoribosyltransferase (*HPRT*) as an endogenous control (FAM reporter dye on the target gene probes, VIC reporter dye on the endogenous control gene probe, Applied Biosystems). TaqMan Fast Universal PCR Master mix and 3 μl of complementary deoxyribonucleic acid (cDNA) were added in a total volume of 10 μl and thermal cycling conditions were; 2 minutes (min) at 50°C; 10 min at 95°C; followed by 40 cycles of 15 seconds (sec) at 95°C and 1 min at 60°C. All samples were analysed in triplicate and the average Ct value was used in all analyses. Relative gene expression (expressed as fold change of mRNA levels in all LOAD tissues relative to the average of mRNA levels in control tissues) was calculated using the 2-ΔΔCt method.

### Enzyme-linked immunosorbent assay (ELISA) with brain tissues

Cerebellum tissues were sonicated on ice in bicarbonate/carbonate solution (100 mM, pH 9.6) and centrifuged at 3000 rpm for 10 min at 4°C and frontal cortex tissues were sonicated in a buffer containing 30 mM EDTA, 250 mM NaCl, 1 mM DTT, 50 mM K2HPO4 (pH 7.2) and centrifuged for 20 min at 4°C. Protein concentration in supernatants was measured with the BCA protein dosage kit (Pierce, Rockford, Il). Indirect ELISA was performed on Costar 96-well EIA/RIA plates (Fisher Scientific, Ottawa, ON) that were first incubated overnight at 4°C with brain protein homogenates and purified synthetic PCSK9 peptide (CRSRHLAQASQELQ) used as standard (437 to 7500 μg/ml), applied in triplicate. The next day, the primary antibody (goat polyclonal PCSK9 antibody, ab28770, Abcam, Cambridge, USA) diluted in a blocking solution (phosphate buffered saline (PBS) 10 mM and bovine serum albumin (BSA) 1%) was added to each well for 2 hours at room temperature. Then, all wells were rinsed with washing buffer (0.1% Tween-20/ 1.0 M Tris base salt) and incubated with the detection antibody in blocking buffer (biotinylated goat antibody, ab6740, Abcam) for another 2 hours. After further washes, the plates were incubated with an alkaline-phosphatase streptavidin solution for 1 hour (Invitrogen Canada Inc., Burlington, ON). Following this incubation, wells were washed and an alkaline phosphatase fluorescent substrate (AttoPhos, Promega, San Luis Obispo, USA) was added to each well for 30 min at 37°C. Fluorescence was detected with a microplate fluorescent reader (FL600, Bio-Tek, Winooski, VT) with a 450 nm excitation filter and 580 nm emission filter. Results are expressed as the mean fold increase of protein concentrations in LOAD cases relative to the average concentration in control subjects ± standard error of mean (SEM). For LDLR protein quantification, we used mouse liver homogenates (sonicated in PBS 10 mM) to produce a standard curve with concentrations ranging from 4 to 135 μg/ml. The primary and detection antibodies used were a rabbit polyclonal LDLR antibody (NB110-57162, Novus Biological, Littleton, USA) and a biotinylated rabbit antibody (ab6720, Abcam), respectively.

### ELISA with CSF samples

Lumbar punctures were performed using a Sprotte 24-gauge atraumatic needle following an overnight fast in volunteers from the PREVENT-AD cohort. All participants from the PREVENT-AD cohort are cognitively normal individuals with a family history of AD-like dementia and are less than 15 years from their siblings’ or parents’ age at symptom onset (minimum of 55 years old). Within 4 hours after lumbar puncture, CSF samples were centrifuged to exclude cells and insoluble material. All CSF samples were then aliquoted and stored at -80°C until needed. PCSK9 protein concentration was measured in undiluted CSF samples with the Quantikine ELISA kit (cat# DPC900 from R&D Systems, Inc. Minneapolis, USA) following the manufacturer’s instructions. APOE, APOB and APOJ (CLU) levels were measured using the apolipoprotein Assay kit (10-plex Magnetic Immunoassays, cat# 12003081 from BioRad, California, USA). CSF samples were diluted 1:500 in sample dilution buffer prior to analysis performed as described by the manufacturer. The CSF AD biomarkers pTau, Tau and Aβ were measured according to the procedures from the BIOMARKAPD consortium of the EU Joint Program in Neurodegenerative Diseases using the validated Innotest enzyme-linked immunosorbent assay kit (pTau cat# 81581, Tau cat# 81579 and Aβ42 cat# 81583, from Fujirebio, Ghent, Belgium) as described before [36]. Data used for this study was collected between September 2011 and August 2017 and archived in PREVENT-AD data release 5.0.

### Genotyping in the PREVENT-AD cohort

Automated DNA extraction from buffy coat samples was performed using the QiaSymphony DNA mini kit (Qiagen, Toronto, Canada). Genotypes were determined with the Illumina Infinium Omni2.5M-8 array (Illumina, San Diege, CA, USA). PLINK tool set [37] (http://pngu.mgh.harvard.edu/purcell/plink/) was used to: 1) filter gender mismatches, 2) filter missingness at both the sample-level (< 5%) and SNP-level (< 5%), 3) assess sample heterozygosity and 4) filter SNPs in Hardy-Weinberg disequilibrium (p>0.001). Reference alleles on GRCh37 coordinates were set according to the forward strand and the final .vcf file was send to Sanger Imputation Service [38] (https://imputation.sanger.ac.uk). Pre-phasing was performed with SHAPEIT2 [39] and PBWT [40] was used for imputation with the 1000 Genomes (phase 3) selected as reference panel. Only post-imputed SNPs with an info score > 0.7 were kept. For *PCSK9* locus, a total of 168 SNPs spanning the genomic region 55 496 039–55 530 526 on GRCh37 coordinates were computed for the expression quantitative trait loci (eQTL) analysis.

### Genotyping in the Quebec Founding Population (QFP) cohort

Genomic DNA samples from Genizon [41] and the Douglas—Bell Canada Brain Bank [42] were extracted from blood or brain tissue using the DNeasy tissue kit (Qiagen, Toronto, Canada) and automated DNA extraction (NA-1000, AutoGen, Holliston, USA). Genotyping was performed with the Illumina 550k Human Quad array (Illumina, San Diego, USA) and quality controlled using PLINK as described in the previous paragraph. *PCSK9* SNPs rs4927193 and rs499718 passed quality controls and did not need to be imputed.

### Alzheimer Disease Neuroimaging Initiative (ADNI) dataset

The ADNI was launched in 2003 as a public-private partnership, led by Principal Investigator Michael W. Weiner, MD. The primary goal of ADNI has been to test whether serial magnetic resonance imaging, positron emission tomography, other biological markers, and clinical and neuropsychological assessment can be combined to measure the progression of MCI and early AD. The ADNI genetic data obtained using the Human 610-Quad BeadChip (Illumina, San Diego, USA) were downloaded from the ADNI website (www.loni.ucla.edu/ADNI). *PCSK9* SNPs rs4927193 and rs499718 passed PLINK quality controls and did not required imputation. For CSF measurements and up-to-date information, see www.adni-info.org.

### Statistical analyses

Comparisons of *PCSK9* and *LDLR* expression levels between LOAD and controls were done using a Student T-test for mRNA levels and a Mann-Whitney U test for protein levels (SPSS 19.0 software). Correlational analyses of CSF PCSK9 levels and other CSF proteins were assessed by linear regression using SPSS 19.0. All genetic association analyses were performed with PLINK tool set [37] (http://pngu.mgh.harvard.edu/purcell/plink/).

## Results

### *PCSK9* is overexpressed in LOAD frontal cortices

**Fig 1:**
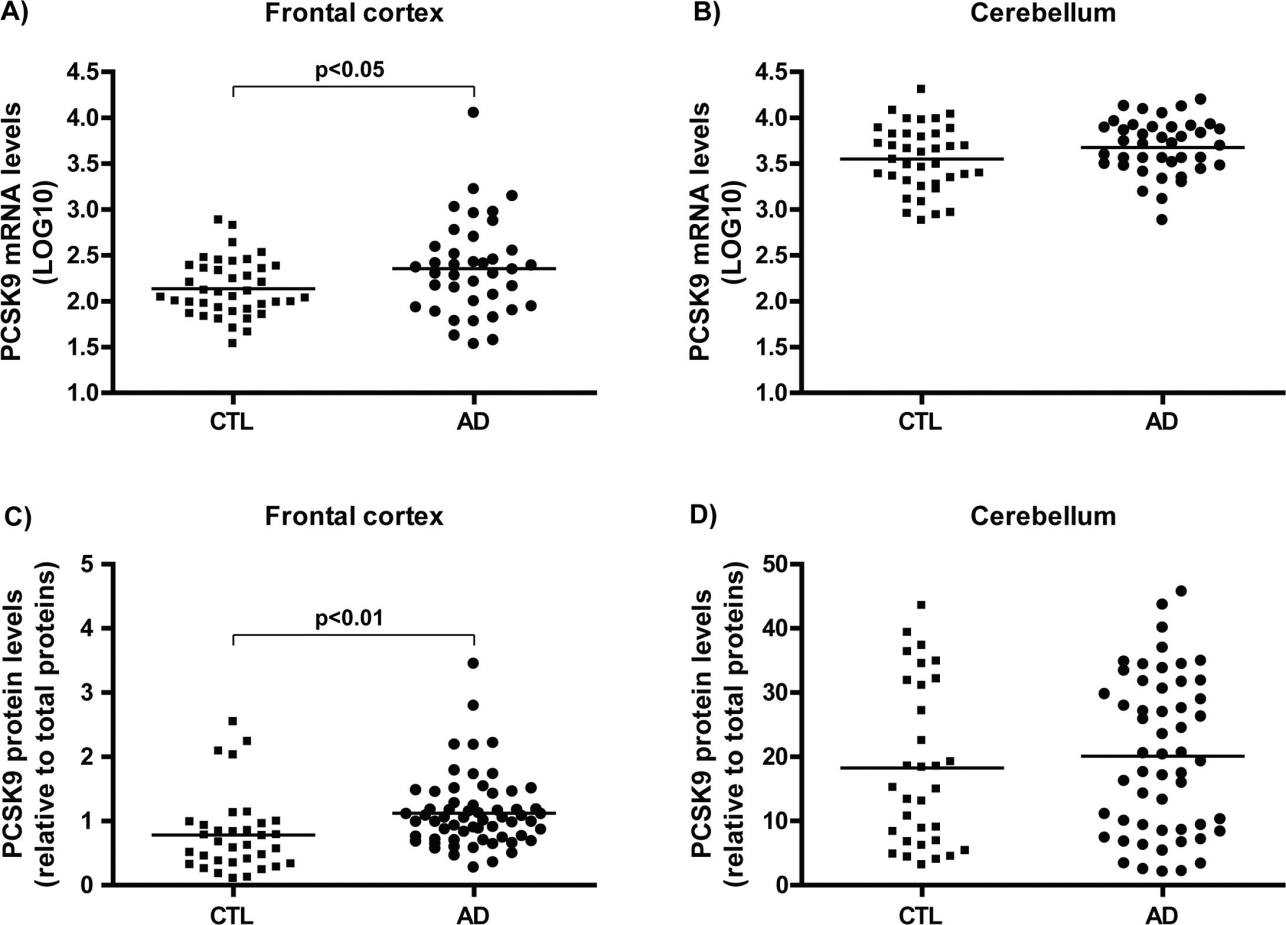
*PCSK9* expression levels in AD compared to control brains. Relative *PCSK9* mRNA levels in A) frontal cortices and B) cerebella of AD and control brains from the QFP quantified by RT-PCR. Relative PCSK9 protein levels in C) frontal cortices and D) cerebella of AD and control brains quantified by ELISA. AD, Alzheimer’s disease; CTL, controls; mRNA, messenger ribonucleic acid; PCSK9, proprotein convertase subtilisin/kexin type 9.

In order to investigate the contribution of *PCSK9* in LOAD, its expression was assessed in the brain of autopsy-confirmed AD cases and age/gender-matched control subjects. mRNA prevalence was assessed by quantitative RT-PCR and *PCSK9* levels in frontal cortices and cerebella of LOAD was compared to control subjects as illustrated in Fig 1A and 1B. *PCSK9* gene expression in frontal cortices was found to significantly differ in AD versus control subjects (Fig 1A, p<0.05) but not in cerebellum area (Fig 1B), a low pathology control brain area. Similarly, ELISA analyses of PCSK9 protein contents revealed increases in frontal cortices of AD patients compared to control subjects (Fig 1C, p<0.01), but not in cerebellum area (Fig 1D).

### *LDLR* transcripts are elevated in LOAD frontal cortices

**Fig 2:**
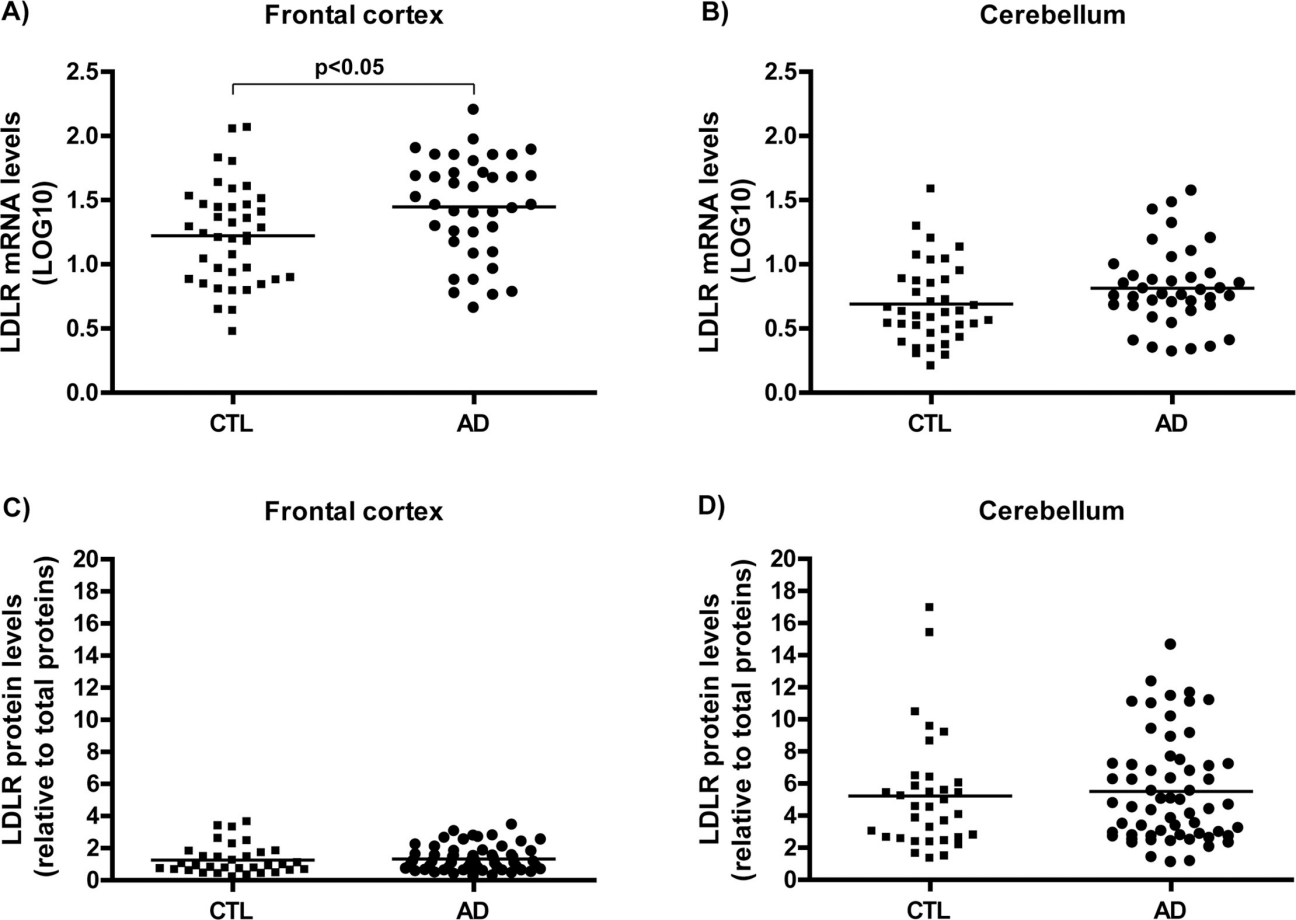
*LDLR* expression levels in AD compared to control brains. Relative *LDLR* mRNA levels in A) frontal cortices and B) cerebella of AD and control brains from the QFP quantified by RT-PCR. Relative LDLR protein levels in C) frontal cortices and D) cerebella of AD and control brains quantified by ELISA. AD, Alzheimer’s disease; CTL, controls; mRNA, messenger ribonucleic acid; LDLR, low density lipoprotein receptor.

Consistent with PCSK9 observations, *LDLR* mRNA prevalence was significantly increased in frontal cortices of LOAD patients relative to age/gender-matched control subjects (Fig 2A, p<0.05) but unchanged in cerebella (Fig 2B). In contrast, LDLR protein levels remained unaffected in both cerebella and frontal cortices (Fig 2C and 2D). This observation is actually consistent with the notion that PCSK9 normally acts as an enhancer of LDLR protein degradation; the more PCSK9 available, the less LDLR detected despite compensatory upregulation of *LDLR* mRNA levels in the same brain area.

### CSF PCSK9 and related apolipoproteins

**Fig 3:**
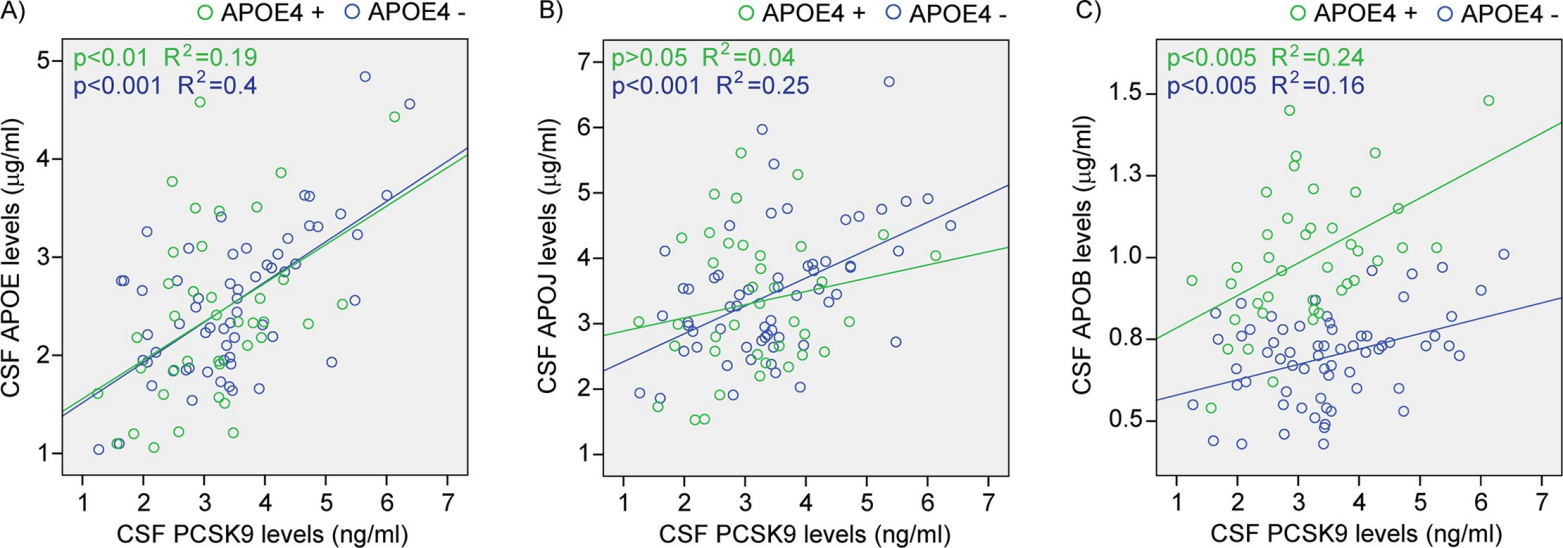
CSF PCSK9 protein levels compared to apolipoproteins in function of *APOE4* allele. PCSK9 and A) APOE, B) APOJ and C) APOB protein levels measured by ELISA in 104 CSF samples of cognitively normal subjects from the PREVENT-AD cohort. APOB, apolipoprotein B; APOE, apolipoprotein E; APOE4, apolipoprotein E (ε4 allele); APOJ, apolipoprotein J; CSF, cerebrospinal fluid; PCSK9, proprotein convertase subtilisin/kexin type 9.

To better understand the relation between PCSK9 and other secreted proteins involved in brain cholesterol metabolism in “at-risk” subjects, we performed parallel CSF measurements of PCSK9 and different apolipoproteins in cognitively normal subjects with a parental history of LOAD. CSF PCSK9 levels correlated strongly with those of APOE, regardless of *APOE4* genotype (all subjects p = 2.4E-09, Fig 3A). For CSF APOJ levels, the correlation with PCSK9 was significant only in *APOE4*- subjects (p = 4.7E-05, Fig 3B). Although APOB is not produced in the central nervous system, small amounts can be detected in the CSF [43, 44] using sensitive ELISA or Luminex assays, as observed in Fig 3C. Not only do APOB levels correlated well with those of PCSK9 (*APOE4*- p = 1.2E-03, *APOE4*+ p = 1.2E-03, Fig 3C), but stratification by *APOE4* genotype reveals marked increases of APOB in *APOE4*+ carriers when compared to non-carriers (p = 1.3E-14, data from Fig 3C). These results indicated that in pre-symptomatic individuals which underwent lumbar punctures on average 15 years before their parent’s LOAD onset, *APOE4* carriers appears to exhibit a compromised blood-brain barrier that facilitate the penetration of small amount of peripheral APOB (presumably from LDL) into the brain. Here, although *APOE4* genotype stratification did not affect APOE’s own protein concentrations in the CSF (p = 0.43, data from Fig 3A), the presence of the *APOE4* allele certainly influenced the metabolism of other cholesterol-related proteins, as seen in Fig 3B.

### CSF PCSK9 levels correlate with AD biomarkers Tau and pTau

**Fig 4:**
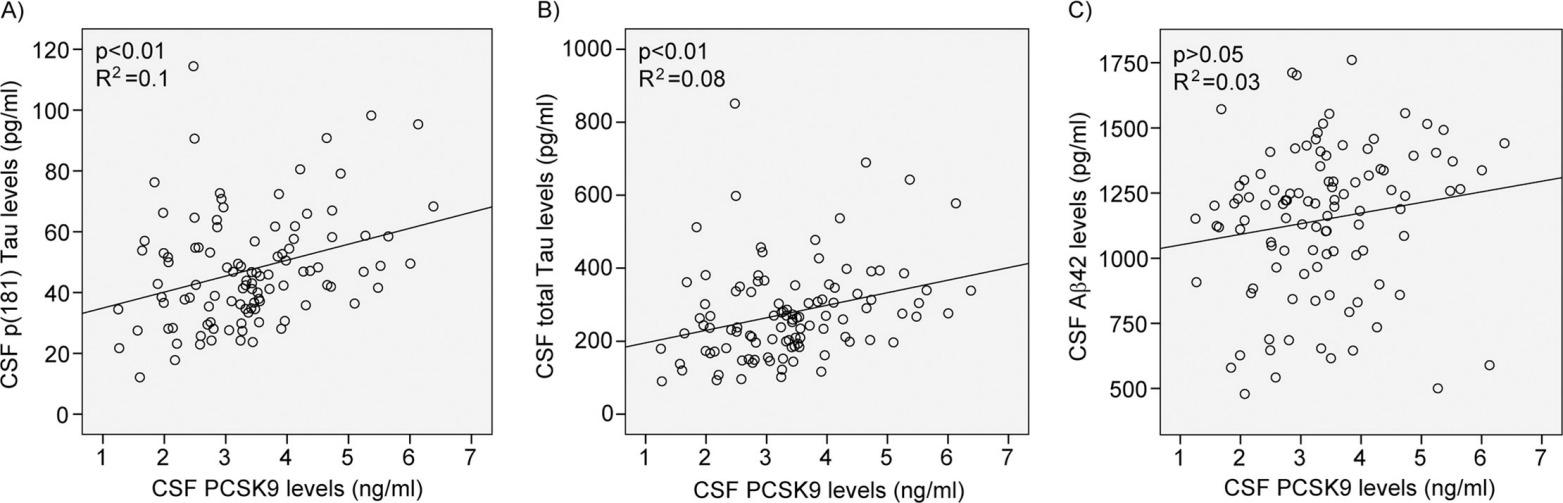
CSF PCSK9 protein levels compared to AD biomarkers. PCSK9 and A) p(181) Tau, B) total Tau and C) Aβ42 protein levels measured by ELISA in 104 CSF samples of cognitively normal subjects from the PREVENT-AD cohort. Aβ42, amyloid beta 42; CSF, cerebrospinal fluid; PCSK9, proprotein convertase subtilisin/kexin type 9; pTau, phosphorylated Tau.

To address the possibility that PCSK9 is indirectly modulating AD pathophysiology, we measured AD biomarkers in the CSF of pre-symptomatic “at-risk” individuals and contrasted each marker to PCSK9 levels. A significant positive correlation was observed between CSF PCSK9 levels and CSF p(181)Tau (p = 0.001, Fig 4A) and total Tau (p = 0.003, Fig 4B). No significant correlation was observed between CSF PCSK9 and Aβ42 levels (Fig 4C). These results suggest that PCSK9 is more likely to influence tau metabolism and neurofibrillary tangles accumulation rather than amyloid plaques deposition, at least in the pre-symptomatic phase of LOAD.

### SNPs associated with CSF PCSK9 levels affect LOAD risk and CSF Tau in females

**Fig 5:**
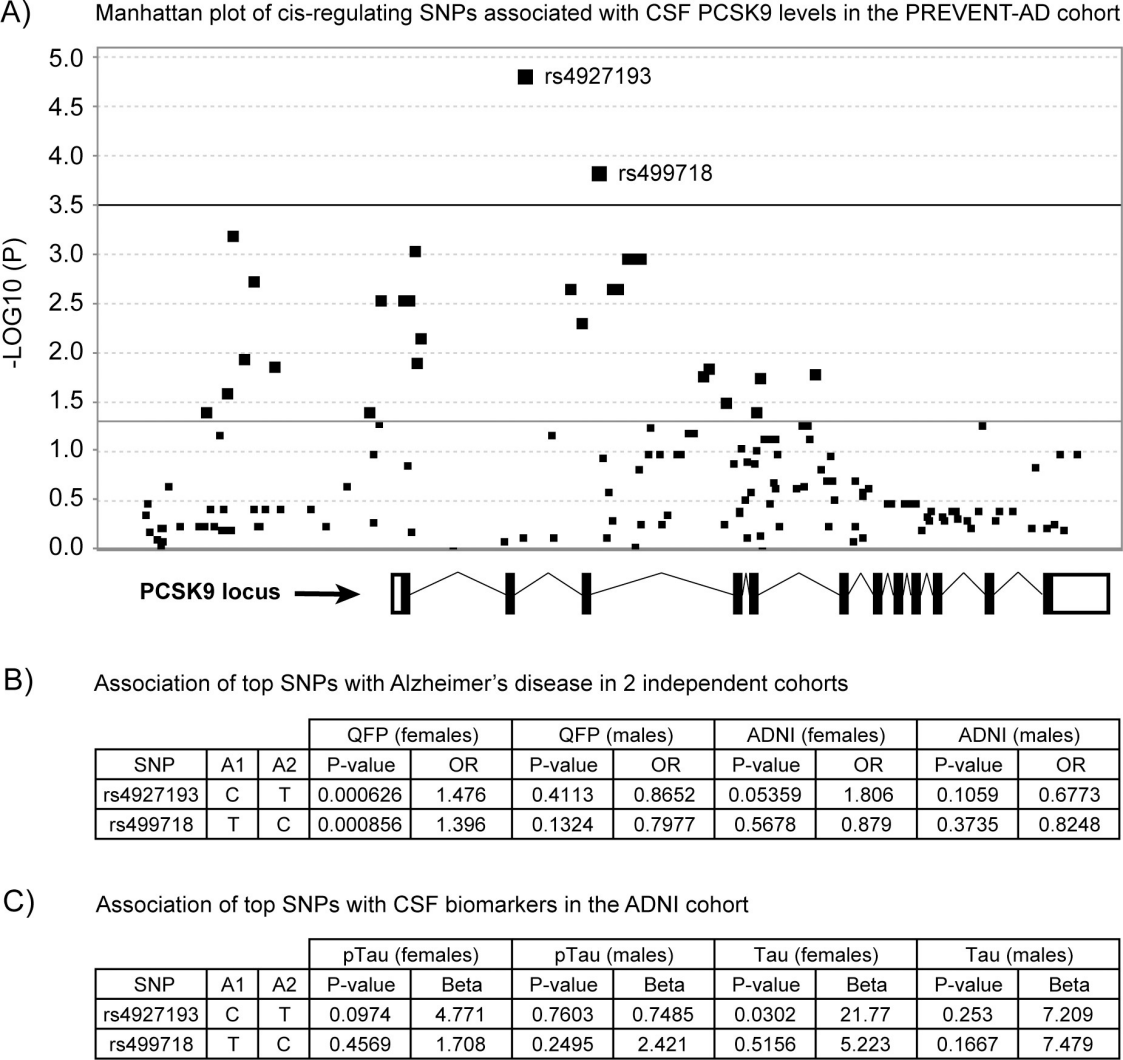
Genetic associations with CSF PCSK9 levels, Alzheimer’s disease risk and specific biomarkers. Genetic associations with A) CSF PCSK9 levels measured by ELISA in 82 cognitively normal subjects for which full genetic data was available. Intronic SNPs rs4927193 and rs499718 reached significance for *PCSK9* locus (-log10(P) for a total of 168 SNPs = 3.5). Genetic associations of rs4927193 and rs499718 with B) Alzheimer’s disease in 1952 subjects from the QFP and 535 subjects from ADNI and C) with pTau and Tau from 384 CSF samples from ADNI according to gender. A1, allele 1; A2, allele 2; ADNI, Alzheimer’s disease Neuroimaging Initiative; CSF, cerebrospinal fluid; OR, odds ratio; PREVENT-AD, pre-symptomatic evaluation of experimental or novel treatments for Alzheimer’s disease; PCSK9, proprotein convertase subtilisin/kexin type 9; pTau, phosphorylated Tau; QFP, Quebec founder population; SNP, single nucleotide polymorphism.

To examine the genetic contribution of *PCSK9* variants on CNS gene expression and their influence on AD pathology, a cis-eQTL study were performed on the *PCSK9* locus. After contrasting CSF PCSK9 levels (data from Figs 3 and 4) and polymorphisms in the *PCSK9* gene locus in PREVENT-AD subjects, two distinct intronic SNPs were found to reach statistical significance threshold (Fig 5A). The latter polymorphisms were then analysed in two independent cohorts comprising 1) of autopsy-confirmed healthy controls and AD subjects from the QFP cohort to assess LOAD risk levels and, 2) of living subjects from the ADNI cohort to assess the effect of genetic stratification on CSF p(181) Tau and Tau concentrations. In the QFP, post-mortem evaluation of AD pathology revealed a significant association with LOAD risk in females only (rs4927193 p = 0.000626 and rs499718 p = 0.000856, Fig 5B). Association between rs4927193 and LOAD risk reached significance in female participants from ADNI (rs4927193 p = 0.05, Fig 5B). To reassess the possible link with tau pathology, *PCSK9* genetic markers were contrasted with CSF pTau and Tau levels from cognitively impaired individuals enrolled in ADNI. In females, a weak association was observed for rs4927193 and CSF pTau (p = 0.0974, Fig 5C) but a significant one was reached with CSF Tau (p = 0.0302, Fig 5C). Regarding this last analysis, note that the mean age and MOCA scores did not differ between males and females.

## Discussion

PCSK9 is a serine protease first described to target hepatic LDLR and to mediate its degradation [45]. Gain-of-function *PCSK9* mutations lead to increased levels of serum LDL cholesterol and loss-of-function mutations prevent the degradation of the hepatic LDLR, resulting in a higher clearance of plasma LDL-cholesterol [46]. PCSK9 is expressed in the brain [47] and is detectable in the CSF of healthy subjects without the typical diurnal pattern of plasma PCSK9 [48]. In neurons, PCSK9 has been shown to degrade LDLR [49] as well as other APOE-binding receptors such as the very low-density lipoprotein receptor (VLDLR), the LDL receptor related protein 1 (LRP1) and the APOE receptor type 2 (APOER2); these receptors mediate the internalization of the cholesterol transported within CSF by HDL-like lipoprotein particles [50]. It is thus conceivable that altered PCSK9 activity in the CNS may contribute to the reported deterioration of brain cholesterol homeostasis observed in LOAD and indirectly, to lipoprotein dysfunction and AD pathophysiology.

Several “loss-of-function” and “gain-of-function” mutations have been described in the *PCSK9* gene [51]. “Loss-of-function” mutations, for example *PCSK9* R46L (rs11591147), were associated with lower circulating LDL-cholesterol levels through increased LDLR levels, resulting in a lower prevalence of peripheral arterial disease and a reduced risk of coronary heart disease [52]. A recent analysis of *PCSK9* R46L and InsLEU mutation frequency distribution in a large cohort of autopsy-confirmed and clinical control and LOAD cases from Eastern Canada failed to show protection in carriers of the loss-of-function mutations [53]. Furthermore, Mendelian randomisation using the *PCSK9* R46L (rs11591147) variant did not find any obvious association with impaired cognitive performance or functional status in 5777 elderly participants enrolled in PROspective Study of Pravastatin in the Elderly at Risk (PROSPER) [54].

In autopsy-confirmed human brains, we report here a significant increase in cortical *PCSK9* gene expression and protein levels in LOAD when compared to age-matched control subjects (Fig 1A and 1C). In the cerebellum, a brain region with minimal pathology, *PCSK9* gene expression and protein levels are not altered in the AD brains (Fig 1B and 1D). Considering the severity of the pathology at the end-stage of the disease, the increased levels are consistent with previous reports of increased *PCSK9* expression in response to neuronal injury such as serum withdrawal-mediated apoptosis where *PCSK9* is markedly upregulated [55] and, in response to transient ischemic stroke in adult mice [9].

In the LOAD brain, the *LDLR* gene expression is up-regulated in the cortical areas but not in the cerebellum (Fig 2A and 2B); presumably in response to neuronal damage and the ensuing compensatory response believed to facilitate synaptic remodelling [56, 57]. In contrast, LDLR protein levels remains unchanged (Fig 2C and 2D), presumably in response to the concomitant induction of PCSK9 in the region; promoting local LDLR degradation.

In the CSF of “at-risk” cognitively normal individuals, CSF PCSK9 protein levels correlate strongly with those of APOE (Fig 3A). The observation is consistent with the notion that PCSK9-mediated reduction of LDLR (and other APOE receptors) attenuates the internalisation of APOE- and APOJ-containing lipoprotein particles, thus leading to increased concentrations in the extracellular space. Of note, the correlation between PCSK9 and APOE protein levels was found to be independent of *APOE4* genotype in pre-symptomatic individuals.

Interestingly, when CSF PCSK9 levels are contrasted with those of APOJ, a significant correlation was observed only in *APOE4*- carriers (Fig 3B). In the CSF, APOJ is normally found in lipid particles that may or not contain APOE [58]. It facilitates lipoprotein endocytosis by binding specifically to LDL receptor related protein 2 (LRP2) [59]; a lipoprotein receptor whose catabolism is not modulated by PCSK9 [60]. It is thus conceivable that only APOE4- lipoprotein particles containing APOJ are affected by the action of PCSK9 toward LDLR, in a dose-dependent manner. Even if lipoproteins containing APOE3 and APOE4 bind LDLR with the same affinity [61, 62], APOE3 was shown to participate in stronger protein-protein interactions in the HDL particle surface compared to APOE4 [63].

*APOE4* genotype is a well-known risk factor for LOAD but its role in the pre-symptomatic phase of the disease is less clear. Here we show that *APOE4* carriers display higher levels of CSF APOB compared with *APOE4* non-carriers (Fig 3C) and both genotypes show statistically significant correlations between CSF PCSK9 and APOB levels. APOB is normally found in periphery as part of the LDL particles and, it is not synthesized in the central nervous system [64]. Its elimination through LDLR binding in the CNS and its subsequent internalisation could likely be modulated by PCSK9 in a manner similar to HDL particles present in the CSF.

In a recent paper, Courtemanche *et al* showed that CSF PCSK9 was elevated in AD and other neurodegenerative diseases compared to controls [65]. When all groups were analysed together, they saw significant positive correlations between CSF PCSK9 and pTau as well as with Aβ42. As pTau levels are known to rise in AD patients and, at the opposite, Aβ42 levels are lowered in AD patients, these results are a priori contradictive. Our results show that CSF PCSK9 correlate positively with both, pTau and Tau, while no correlation was found with Aβ42 in pre-symptomatic “at-risk” subject (Fig 4).

*In vitro*, *PCSK9* overexpression was shown to result in the reduction of endogenous BACE1 levels, also known as beta-site amyloid precursor protein cleaving enzyme 1, with a decrease of both immature and mature forms [11]. In contrast, the down-regulation of *PCSK9* by siRNA completely normalized the levels of BACE1 [11]. In addition, *Pcsk9* knockout mice showed higher levels of BACE1 and Aß in the neocortex [11]. However, more recent independent *in vivo* work reported a lack of effect of *PCSK9* overexpression, or deletion (knockout), on the levels of BACE1 in the mouse brain, with a concomitant lack of effect also on Aß levels [12]. The authors concluded that PCSK9 has no effect on BACE1 enzyme *in vivo*.

In terms of gene regulation, both *PCSK9* [20] and *BACE1* [66] were shown to be upregulated by SREBF2 via sterol-regulatory element in their respective promoter. In rats fed a high fat diet, the observed reduction of free cholesterol was suggested to activate SREBF2 and BACE1 [66]. Similarly, apoE (-/-) mice fed a high fat diet showed increased in *Pcsk9* and *Bace1* expression levels [67]. It is therefore possible that PCSK9 effect on BACE1 depends on cholesterol levels and SREBF2 activation.

Interestingly, a compound isolated from a Chinese herb was shown to decrease PCSK9 mRNA and protein levels [68] while reducing Aβ levels [69]. After a 3-month treatment with this alkaloid named berberine, a 29% reduction of serum cholesterol, 35% reduction of triglyceride and 25% reduction of LDL-c were achieved in hypercholesterolemic participants [70]. Since berberine can cross the blood-brain barrier [71] and has other neuroprotective properties, it was suggested to be an agent to combat AD [72]. These findings support a neuroprotective effect of PCSK9-lowering therapies, strengthening the lack of association with impaired cognition [73].

Our results suggest that PCSK9 is definitively involved with Tau pathology in the earlier (pre-symptomatic) stage of the disease and may eventually modulate the amyloid pathway via BACE1 as disease pathology expand. This will be examined in future studies as many of our asymptomatic subjects are bound to convert into mild cognitively impaired, and eventually in LOAD over time.

Although we could not find any beneficial protective effect of “loss of function” *PCSK9* R46L and InsLEU mutations in our recent case-control study [53], it prompted us to further examine the variants distribution in the *PCSK9* gene: leading to the identification of two interesting SNPs, namely rs4927193 and rs499718, located in *PCSK9* intron 2 and 3 respectively. These variants were found to strongly associate with CSF PCSK9 levels in the CSF of our asymptomatic subjects. The association between rs4927193 C allele and AD risk in females from the QFP cohort (p<0.001, O.R. 1.46) is suggestive of a gender-specific risk allele in this population. Replication was achieved in the ADNI cohort (OR 1.806; p = 0.053) female subgroup: the association was not found in males from the either QFP or ADNI cohorts. Interestingly, a similar gender-specific difference was observed when stratifying CSF pTau and Tau values by *PCSK9* rs4927193 polymorphism (Fig 5C). In contrast to circulating PCSK9 levels which are generally higher in females compared to males, particularly after the age of 50 [74, 75], such an effect is not observed in the CSF of PREVENT-AD participants (PCSK9 mean CSF levels ± SEM in females: 3.35 ± 0.13 ng/ml and males: 3.43 ± 0.21 ng/ml).

The female specificity of the *PCSK9* variant which associates with AD risk is certainly not unique, particularly in genes directly involved in lipid metabolism. *APOE* (extracellular cholesterol transport), *ABCA1* (intracellular cholesterol transport) and *HMGCR* (cholesterol synthesis) all exhibit female-specificity for their AD risk/protection associations [16, 76, 77].

Our results support current knowledge on the functions of PCSK9 in the central nervous system and its possible involvement in AD pathophysiology. Even though a direct mechanistic link between PCSK9 levels and total Tau and phospho-Tau has not been established, the strong positive correlations that exist between those CSF biomarkers in “at-risk” subjects may indicate a subclinical interaction that might increase the risk of cognitive decline.

## Conclusion

PCSK9 appears to play an active role in the pathophysiology of LOAD both in the pre-symptomatic and symptomatic phases of the disease. The *PCSK9* risk allele rs4927193 associated with LOAD was found to affect both PCSK9 levels and, tau-related CSF biomarkers in a gender-specific manner not unlike other lipid associated genes known to be involved in LOAD risk and pathology.

## Abbreviations

Aβ: amyloid beta
Aβ42: amyloid beta 42
ABCA1: ATP binding cassette subfamily A member 1
ABCA7: ATP binding cassette subfamily A member 7
ABCG1: ATP binding cassette subfamily G member 1
AD: Alzheimer’s disease
ADNI: Alzheimer’s disease Neuroimaging Initiative
APOB: apolipoprotein B
APOE: apolipoprotein E
APOE4: apolipoprotein E (ε4 allele)
APOJ: apolipoprotein J
APP: amyloid beta precursor protein
BACE1: beta-secretase 1
BCA: bicinchoninic acid
BIN1: bridging integrator 1
BSA: bovine serum albumin
cDNA: complementary deoxyribonucleic acid
CNS: central nervous system
CSF: cerebrospinal fluid
CTL: controls
DNA: deoxyribonucleic acid
DTT: dithiothreitol
EDTA: ethyleneaminetetraacetic acid
ELISA: enzyme-linked immusorbent assay
eQTL: expression quantitative trait loci
HDL: high-density lipoprotein
HMGCR: 3-hydroxy-3-methylglutaryl-CoA reductase
HPRT: hypoxanthine phosphoribosyltransferase
K2HPO4: potassium phosphate dibasic
LDL: low-density lipoprotein
LDLR: low-density lipoprotein receptor
LOAD: late onset Alzheimer’s disease
LRP1: LDL receptor related protein 1
LRP2: LDL receptor related protein 2
MCI: mild cognitive impairment
mRNA: messenger ribonucleic acid
NaCl: sodium chloride
OR: odds ratio
PBS: phosphate buffered saline
PCSK9: proprotein convertase subtilisin/kexin type 9
PICALM: phosphatidylinositol binding clathrin assembly protein
PREVENT-AD: pre-symptomatic evaluation of experimental or novel treatments for Alzheimer’s disease
pTau: phosphorylated Tau
QFP: Quebec founder population
RNA: ribonucleic acid
RT-PCR: real-time polymerase chain reaction
SEM: standard error of the mean
siRNA: small interfering ribonucleic acid
SNP: single nucleotide polymorphism
SORL1: sortilin related receptor 1
VLDLR: very low-density lipoprotein receptor
